# Combining Fine Mapping, Whole-Genome Re-Sequencing, and RNA-Seq Unravels Candidate Genes for a Soybean Mutant with Short Petioles and Weakened Pulvini

**DOI:** 10.3390/genes13020185

**Published:** 2022-01-21

**Authors:** Keke Kong, Mengge Xu, Zhiyong Xu, Ripa Akter Sharmin, Mengchen Zhang, Tuanjie Zhao

**Affiliations:** 1 National Center for Soybean Improvement, Key Laboratory of Biology and Genetics and Breeding for Soybean, Ministry of Agriculture and Rural Affairs, State Key Laboratory of Crop Genetics and Germplasm Enhancement, Nanjing Agricultural University, Nanjing 210095, China; 2017201030@njau.edu.cn (K.K.); 2017201075@njau.edu.cn (M.X.); 2016201025@njau.edu.cn (Z.X.); ripa.sharmin@gmail.com (R.A.S.); 2 Department of Botany, Jagannath University, Dhaka 1100, Bangladesh; 3 North China Key Laboratory of Biology and Genetic Improvement of Soybean, Ministry of Agriculture and Rural Affairs, National Soybean Improvement Center Shijiazhuang Sub-Center, Laboratory of Crop Genetics and Breeding of Hebei, Cereal & Oil Crop Institute, Hebei Academy of Agricultural and Forestry Sciences, Shijiazhuang 050000, China

**Keywords:** soybean, short petioles and weakened pulvini mutant, fine mapping, whole-genome re-sequencing, candidate genes, RNA-seq

## Abstract

A short petiole is an important agronomic trait for the development of plant ideotypes with high yields. However, the genetic basis underlying this trait remains unclear. Here, we identified and characterized a novel soybean mutant with short petioles and weakened pulvini, designated as *short petioles and weakened pulvini* (*spwp*). Compared with the wild type (WT), the *spwp* mutant displayed shortened petioles, owing to the longitudinally decreased cell length, and exhibited a smaller pulvinus structure due to a reduction in motor cell proliferation and expansion. Genetic analysis showed that the phenotype of the *spwp* mutant was controlled by two recessive nuclear genes, named as *spwp1* and *spwp2*. Using a map-based cloning strategy, the *spwp1* locus was mapped in a 183 kb genomic region on chromosome 14 between markers S1413 and S1418, containing 15 annotated genes, whereas the *spwp2* locus was mapped in a 195 kb genomic region on chromosome 11 between markers S1373 and S1385, containing 18 annotated genes. Based on the whole-genome re-sequencing and RNA-seq data, we identified two homologous genes, *Glyma.11g230300* and *Glyma.11g230600*, as the most promising candidate genes for the *spwp2* locus. In addition, the RNA-seq analysis revealed that the expression levels of genes involved in the cytokinin and auxin signaling transduction networks were altered in the *spwp* mutant compared with the WT. Our findings provide new gene resources for insights into the genetic mechanisms of petiole development and pulvinus establishment, as well as soybean ideotype breeding.

## 1. Introduction

The geometrical and topological organization of components of various plant types and shapes defines the architecture of plants [1]. For aboveground plant parts, this mainly includes stem growth habit, branching pattern, plant height, internode length, petiole length, and leaf size as well as shape in soybean. Among these components, petiole length is an important factor that influences canopy architecture that directly affects light interception efficiency, photosynthetic efficiency, and, ultimately, yield. The short petiole trait is potentially useful for improving the per unit yield by improving planting density and altering the canopy profile in soybean [2,3,4]. For instance, stable GmMYB14-overexpressing transgenic soybean plants displayed a compact plant architecture with short petioles that can be cultivated under higher density, thereby showing increased yields [5].

The genetic regulation mechanism for the development of the leaf petiole is controlled by a complex regulatory network. Although the basic form of a leaf is clearly divided into the leaf blade and leaf petiole, the development of both is closely related. The *LEAFY PETIOLE* (*LEP*) gene is expressed in leaf primordia and developing leaf blades, and overexpression of the *LEP* gene is sufficient to transform the proximal part of the leaf from a petiole into a leaf blade [6]. Similar phenotypes were also found in the *blade-on-petiole1-1* (*bop1-1*) mutant, which has leaflet-like structures on leaf petioles [7]. The *BOP1* gene encodes a NONEXPRESSOR OF PR GENES1 (NPR1)-like protein that regulates leaf differentiation in a proximal–distal manner by negatively regulating the expression of class I *knotted-like homeobox* (*knox*) genes [8]. Mutations in the Arabidopsis *JAGGED* (*JAG*) gene can cause absent petioles or develop blade tissue along petiole edge phenotypes [9,10]. These results suggest that the *LEP*, *BOP* and *JAG* genes are important not only for leaf development but also for petiole development. Recently, Favero et al. [11] demonstrated that the AT-hook motif nuclear-localized (AHL) transcription factors repress leaf petiole elongation by antagonizing the growth-promoting PHYTOCHROME-INTERACTING FACTORS (PIFs). In soybean, however, few genes were reported to control petiole length, and little is known about the regulatory mechanisms of petiole growth and development.

Nyctinasty, a term used to describe the closure of leaves during the night, is a common and characteristic phenomenon found in Fabaceae, Maranthaceae, and Oxalidaceae species [12]. This movement is triggered by the pulvinus, a joint-like thickening located at the base of the leaf or leaflet [13]. Two functionally and positionally antagonistic groups of motor cells, the adaxial flexor and the abaxial extensor, exist in the pulvinus [14], and the motor cells are responsible for leaf movement through changes in turgor pressure [15,16,17,18]. Therefore, the pulvinus plays a critical role in leaf movement. Several mutants with defects in pulvini have been identified in legume species, such as the *apulvinic*/*apu* mutant in *Pisum sativum* [19], the *sleepless*/*slp* mutant in *Lotus japonicus* [20], and the *petiolule-like pulvinus*/*plp* or *elongated petiolule1*/*elp1* mutant in *Medicago truncatula* [21,22]. These mutants formed petiolule-like structures at the base of leaflets instead of pulvini and the motor cells were replaced by elongated petiole-like epidermal cells. More importantly, these genes, *viz.*, *APU*, *SLP1*, and *PLP*/*ELP1*, all encode a conserved higher plant-specific LATERAL ORGAN BOUNDARIES (LOB) domain-containing protein, suggesting that the determination of pulvinus identity in legumes is likely to be regulated by a conserved genetic network [16,23]. Soybean, similar to many Fabaceae species, has a pulvinus at the base of the petiole. The *GmILPA1* (*Glycine max Increased Leaf Petiole Angle 1*) gene is critical for the structure establishment of pulvinus and functions by promoting motor cells growth and division in the pulvinus [24]. So far, there are few reports on the soybean pulvinus and its developmental determination remains largely unknown. 

In the present study, we identified a novel soybean mutant with short petioles and weakened pulvini, designated as *short petioles and weakened pulvini* (*spwp*). To isolate the target genes, we integrated map-based cloning, whole-genome re-sequencing, and RNA-seq strategies. We identified two important candidate genes, *Glyma.11g230300* and *Glyma.11g230600*, that both encode protein-kinase-domain-containing proteins, which possess nucleoside acid differences in coding sequences and are significantly differentially expressed between the *spwp* mutant and the wild type. Furthermore, RNA-seq analysis revealed that many differentially expressed genes (DEGs) involved in cytokinin and auxin signaling transduction networks were closely associated with the formation of the *spwp* mutant. This study provides a new genetic resource for ideal plant type breeding and enriches the understanding of the molecular basis underlying the short petiole formation and pulvinus development.

## 2. Materials and Methods

### 2.1. Plant Materials and Growth Conditions 

The soybean *spwp* mutant was derived from NJ90L-1SP [25]. Seeds of soybean cultivars NanNong1138-2 (NN1138-2) and KeFeng1 (KF1) were obtained from Nanjing Agricultural University, Jiangsu Province, China. For genetic analysis and gene mapping, the *spwp* mutant, as the male parent, was crossed with NN1138-2 and KF1 to construct different genetically segregating populations. 180 F_2_ wild-type individuals of a KF1 × *spwp* cross were randomly harvested to obtain a F_2:3_ line population for progeny tests and gene fine-mapping in the following year. NN1138-2, KF1, *spwp*, and their F_1_, F_2_, and F_2:3_ (derived from the F_2_ of a KF1 × *spwp* cross) populations were grown in the normal growing season at the Jiangpu Agricultural Experiment Station of Nanjing Agricultural University. From a line with a separation ratio of 3:1, a pair of near-isogenic lines, *viz.*, the WT (wild type) and MT (mutant type), was constructed through selfing to advance the generations. The near-isogenic lines were planted in greenhouse conditions at Nanjing Agricultural University (Nanjing, China) to study their phenotypic characteristics.

### 2.2. Histological Analysis

The middle part of the pulvini and petioles at the third node, counted from the top to the bottom, were collected from the WT and the *spwp* mutant plants. All samples were fixed for one week at 4 °C in formalin-aceto-alcohol (FAA) fixing solution (50% alcohol, 5% glacial acetic acid, 5% formalin, and 5% glycerine). The tissues were prepared for paraffin sectioning following the previously described method [24]. Samples were sectioned with a microtome (Leica, RM2245) at a 2 μm thickness and then stained with 1% safranin O/0.5% Fast Green; sections were observed and photographed with a microscope (OLYMPUS BX53). Image J software was used to measure the petiole cell length. 

### 2.3. Inheritance Analysis and Molecular Mapping with Simple Sequence Repeat (SSR) Markers

At the R3 stage (a reproductive stage with ~5 mm long young pods at one of the four uppermost nodes on the main stem) the phenotypes of individuals in the F_2_ and F_2:3_ segregating populations were investigated by visual inspection, and the number of mutant-type and wild-type plants was then recorded. A Chi-square (χ^2^) test was used to analyze the segregation ratio of alleles with the expected ratio at a significance threshold of *p*-value > 0.05 (χ^2^ < 3.84).

Genomic DNA was extracted using a DNAquick Plant System Kit (TIANGEN, DP321) following the standard protocol. Bulk segregant analysis (BSA) was applied to rapidly identify SSR markers potentially linked to the mutated genes [26]. A total of 1015 SSR markers uniformly distributed on the 20 chromosomes were used in the BSA analysis. Among KF1 × *spwp* F_2_ populations, an equal amount of DNA from each of 10 wild-type plants and 10 mutant-type plants formed a wild-type DNA pool and a mutant-type DNA pool, respectively. Primer sequences of SSR markers were obtained from the SoyBase website (http://www.soybase.org, accessed on 10 October 2021) or collected from Song et al. [27]. The PCR amplification program was 95 °C for 3 min followed by 34 cycles of 95 °C for 15 s, 56 °C for 15 s, and 72 °C for 1 min, with final incubation at 72 °C for 5 min before being held at 4 °C. The polymerase chain reaction (PCR) amplification products were separated on 8 % non-denaturing polyacrylamide gels that were stained with 1 g L^−1^ AgNO_3_ for 15 min followed by 16 g L^−1^ NaOH plus 1.1 mL CH_3_OH for 10 min before being visualized under an LED light box. The primers used in the fine-mapping are listed in Tables S1 and S2.

### 2.4. DNA Library Construction and Whole-Genome Re-Sequencing

Genomic DNA was extracted from 5 plants of the WT and MT (a pair of near-isogenic lines) and then equally pooled to generate a WT DNA pool and an MT DNA pool, respectively. A total of 1.5 μg of genomic DNA for each pool was used to construct their individual sequencing library using a Truseq Nano DNA HT Sample Preparation Kit (Illumina, San Diego, CA, USA), following the manufacturer’s recommendations. The final libraries constructed above were sequenced by the Illumina HiSeq4000 platform, and 150 bp paired-end reads were generated with an insert size of around 350 bp. To make sure reads were reliable and without artificial bias in the following analysis, raw reads with ≥10% unidentified nucleotides (N), with > 50% bases having phred quality < 5, or with > 10 nt aligned to the adapter, allowing ≤ 10% mismatches were removed to obtain clean reads. In addition, we trimmed putative PCR duplicates generated by PCR amplification in the library construction process. The clean reads were aligned to the soybean reference genome Williams 82 (*Glycine max Wm82.a4. v1*) using Burrows–Wheeler Aligner (BWA, v. 0.7.10; settings: mem-t 4-k 32-M-R) [28]. Alignment files were converted into BAM files using SAMtools software [29]. Variant calling was performed by using the Unified Genotyper function in GATK v. 3.8 software [30]. The single-nucleotide polymorphisms (SNPs) were obtained by using the Variant Filtration parameter in GATK (settings: —filterExpression “QD < 4.0 || FS > 60.0 || MQ < 40.0 “, -G_filter “GQ < 20”, —cluster WindowSize 4). InDel was filtered via the Variant Filtration parameter (settings: —filter Expression “QD < 4.0 || FS > 200.0 ||Read PosRankSum < −20.0 || Inbreeding Coeff < −0.8 ”). SnpEff 4.3t was then applied to annotate all the variants [31]. Theoretically, the genotype of the causal SNPs allele should be completely present in the MT DNA pool, that is to say no reads containing variant target SNP loci should be present in the WT DNA pool. In the present study, an average SNP index of the SNPs in each genomic interval was calculated using a sliding window analysis with a 2 Mb window size and a 50 kb increment. The delta SNP index = SNP index (MT) − SNP index (WT).

### 2.5. RNA Isolation and Quantitative Real-Time RT-PCR Analysis

Total RNAs from leaf petioles and pulvini were extracted using an RNAprep Pure Plant Kit (TIANGEN, Beijing, China) according to the manufacturer’s instructions. Approximately 2 μg of RNA was then reverse-transcribed into first-strand cDNA using a HiScript II Q RT SuperMix for qPCR (+gDNA wiper) Kit (Vazyme, Nanjing, China), following a standard protocol. Quantitative real-time RT-PCR (qRT-PCR) was performed using a ChamQ SYBR qPCR Master Mix (Vazyme, China) on a Roche LightCycler480 PCR system, following the manufacturer’s instructions. The PCR cycling conditions were 95 °C for 30 s followed by 40 cycles of 95 °C for 10 s and 60 °C for 30 s. Three biological replicates with three technical replicates were used for qRT-PCR assays. The soybean housekeeping gene *GmActin11* (*Glyma.18G290800*) was used as an internal reference to normalize all data. The primers used for qRT-PCR were designed by National Center for Biotechnology Information database (NCBI) Primer BLAST and listed in Table S3.

### 2.6. RNA-Seq analysis

The near-isogenic lines (WT and MT) were grown in a greenhouse for RNA-seq analysis. The stem tips of the WT and MT were individually collected, placed in liquid nitrogen, and stored at −80 °C. Total RNA was isolated using the TRIzol reagent (Invitrogen, Carlsbad, CA, USA). RNA integrity was assessed using an RNA Nano 6000 Assay Kit of a Bioanalyzer 2100 system (Agilent Technologies, Santa Clara, CA, USA). The RNA samples were prepared and submitted to the Novogene Biotech Company (Beijing, China) for sequencing. Raw paired-end reads were first filtered with Fastp software to obtain high-quality clean data and then aligned to the *Glycine max Wm82.a4. v1* using Hisat2 v2.0.5 software. Gene expression (FPKM, fragments per kilobase of transcript per million fragments mapped) levels were calculated based on the length of the gene and read count mapped to this gene. DEGs were identified using the criteria *p*-value ≤ 0.05 and |log2 (Fold Change)| ≥ 1. Gene Ontology (GO) and Kyoto Encyclopedia of Genes and Genomes (KEGG) enrichment analysis of DEGs was implemented by the clusterProfiler R package.

## 3. Results

### 3.1. Phenotypic Characterization of the Spwp Mutant

**Figure 1:**
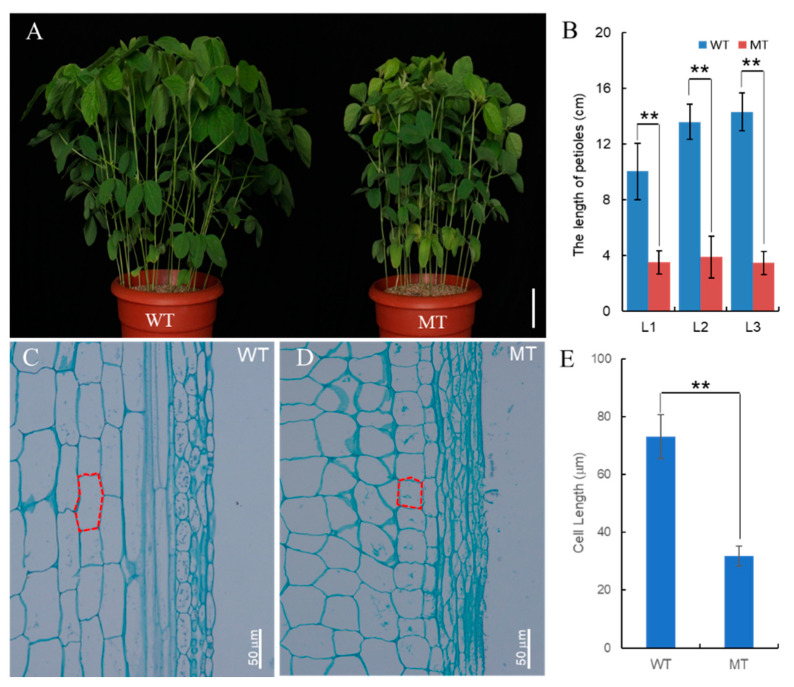
Phenotype comparison between wild-type (WT) and *spwp* mutant-type (MT) plants. (**A**): Phenotype appearance of whole plants of the WT (left) and MT (right). The scale bar is 10 cm. (**B**): Comparison of petiole length from the first to third leaf position between the WT and MT. L1, L2, and L3 represent the first to third leaf petioles from the top to the bottom, respectively. (**C**,**D**): Longitudinal sections on the middle part of the petioles at the third node, counted from the top to the bottom of the WT (**C**) and MT (**D**) plants. The scale bars are 50 μm. (**E**): Comparison of the single-cell length of the leaf petioles between the WT and MT. The result represents the average value of five consecutive cells in a single longitudinal cell file. Significant differences were determined using the Student’s *t*-test: ** *p* < 0.01. All data are given as mean ± SD.

The phenotypes of the wild-type (WT) and the *spwp* mutant-type (MT) plants in the greenhouse are shown in Figure 1A. Compared with the WT, the plant type of the *spwp* mutant is more compact and displays a significant decrease in the length of the petioles. Taking the petiole length from the first to the third leave (from the top to the bottom) as an example, the petiole length was reduced from (10.0 ± 2.0) cm, (13.6 ± 1.3) cm, and (14.3 ± 1.4) cm in the WT to (3.5 ± 0.8) cm, (3.9 ± 1.5) cm, and (3.5 ± 0.8) cm in the MT plants, respectively (Figure 1B). To elucidate the mechanism of short petiole formation at the cytological level, longitudinal sections of petioles were performed. In the MT plants, the cell length was significantly shorter than in the WT ones (Figure 1C–E). This result suggests that the decrease in cell elongation is mainly attributed to the shortened petioles in mutant plants.

**Figure 2:**
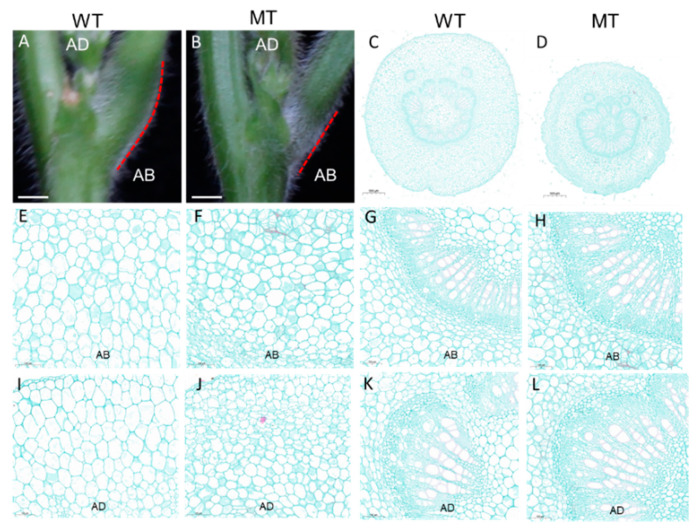
Cross-sectional microstructure of pulvini in wild-type (WT) and *spwp* mutant-type (MT) plants. A and B: Phenotypic characterization of pulvini in WT (**A**) and MT (**B**) plants, respectively. The scale bars are 1 cm. (**C**,**D**): Transection of pulvini in the WT (**C**) and MT (**D**), respectively. The scale bars are 500 μm. (**E**–**L**): Partial magnifying views of the transection of pulvini in the WT (**E**,**G**,**I**,**K**) and MT (**F**,**H**,**J**,**L**). The scale bars are 100 μm. (**E**,**F**) indicate the motor cells of the WT and MT on the abaxial side (AB), respectively. (**I**,**J**) indicate the motor cells of the WT and MT on the adaxial side (AD), respectively. (**G**,**H**) indicate the vascular cylinder of the WT and MT on the abaxial side, respectively. (**K**,**L**) indicate the vascular cylinder of the WT and MT on the adaxial side, respectively.

The pulvinus of the MT was significantly smaller than that of the WT, and the MT did not have the knee-like structure at the base of short petioles that was present in the WT (Figure 2A,B). By comparing the anatomical structure of pulvini (Figure 2C,D) we found that there were fewer and smaller motor cells on both the abaxial side (AB) and adaxial side (AD) in the MT than in the WT (Figure 2E,F,I,J). However, the xylem area of the MT plants was larger than that of the WT, both on the AD and AB (Figure 2G,H,K,L). These observations indicated that cell expansion and proliferation were also affected in the pulvini of the *spwp* mutant plants compared with the WT.

The leaf petiole angle is mainly controlled by the pulvinus [32,33]. To investigate whether the petiole angle of the MT was affected, we measured the petiole angle from the third leaf position to the fifth leaf position (from the top to the bottom) and found that the petiole angle of the MT was slightly increased in comparison to the WT, but the difference was not significant (Figure S1). Apart from the differential phenotypes mentioned above, the nyctinastic motions of pulvini and leaflets were significantly diminished in the *spwp* mutant compared to the wild-type plants.

### 3.2. Genetic Analysis of the Spwp Mutant

To analyze the inheritance pattern of the *spwp* mutant, we crossed *spwp* with NN1138-2 and KF1, respectively. All F_1_ plants of crosses NN1138-2 × *spwp* and KF1 × *spwp* showed a similar phenotype to the wild type, indicating that the *spwp* mutant is recessive. In the F_2_ population of cross KF1 × *spwp*, among the 692 plants only 42 showed the *spwp* mutant phenotype. The ratio of wild-type plants relative to mutant-type plants corresponded to the expected 15:1 segregation ratio for two recessive genes (χ^2^ = 0.02, *p* = 0.87) (Table 1). However, in the case of the NN1138-2 × *spwp* cross, the phenotypic segregation ratio of wild-type relative to mutant-type plants was 420:120, which did not fit the expected ratio of 15:1 but did fit a ratio of 3:1 (χ^2^ = 2.22, *p* = 0.14) (Table 1). We randomly selected 180 F_2_-dominant individuals derived from the KF1 × *spwp* cross to perform the progeny test. In the F_2:3_ lines, the ratio of segregating to non-segregating lines was 93:87, which fitted an expected ratio of 8:7 (χ^2^ = 0.2, *p* = 0.65). These results indicate that the short petioles and weakened pulvini traits of the *spwp* mutant are controlled by two recessive genes, tentatively designated as *spwp1* (*short petioles and weakened pulvini 1*) and *spwp2* (*short petioles and weakened pulvini 2*). These results also imply that the NN1138-2 genome contains either of those two homozygous recessive genes or alleles.

### 3.3. Fine Mapping of the Spwp1 and Spwp2 Genes

**Figure 3:**
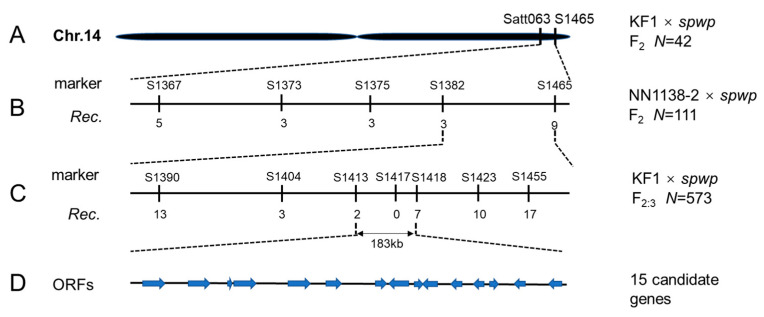
Fine-mapping of the *spwp1* locus. (**A**): the *spwp1* locus was initially mapped by single-sequence repeat (SSR) markers Satt063 and S1465 on chromosome (Chr.) 14 using 42 mutant plants derived from the F_2_ population of KF1 × *spwp*. (**B**): the *spwp1* locus was further mapped to a region between SSR markers S1382 and S1465 using theF_2_ population of NN1138-2 × *spwp*. (**C**): the position of the *spwp1* locus was finally narrowed down to a genomic region of 183 kb between SSR markers S1413 and S1418 using the F_2:3_ population of KF1 × *spwp*. (**D**): Fifteen predicted open reading frames (ORFs) were located in this region according to the Williams 82 reference genome (*Glycine max Wm82.a4.v1*). The numerals below the markers indicate the number of identified recombinants (*Rec.*).

**Figure 4:**
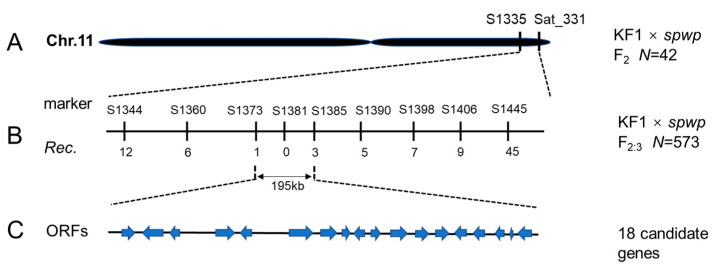
Fine-mapping of the *spwp2* locus. (**A**): the *spwp2* locus was initially mapped by single-sequence repeat (SSR) markers S1335 and Sat_331 on chromosome (Chr.) 11 using the F_2_ population of KF1 × *spwp*. (**B**): the position of the *spwp2* locus was finally delimited to a 195 kb region between SSR markers S1373 and S1385. The numerals below the markers indicate the number of identified recombinants (*Rec.*). (**C**): eighteen predicted open reading frames (ORFs) existed in the 195 kb fine-mapping interval according to the Williams 82 reference genome (*Glycine max Wm82.a4.v1*).

To locate the target genes, a total of 1015 SSR markers were used to screen for polymorphisms between the wild-type and mutant-type DNA pools derived from the F_2_ population of the KF1 × *spwp* cross. We found that three SSR markers, i.e., Satt063, Satt560, and Satt726, on chromosome 14 (linkage group B2), and another three SSR markers, i.e., Satt359, Sat_331, and BE801538, on chromosome 11 (linkage group B1), showed polymorphisms. All 42 F_2_-mutant individuals were used for the further identification of linkage groups and preliminary mapping. New SSR markers from Song et al. [27] were synthesized to determine the mapping regions. After genotyping, the *spwp1* gene was mapped between the SSR markers Satt063 and S1465 on chromosome 14 (Figure 3A); the *spwp2* gene was mapped between the SSR markers S1335 and Sat_331 on chromosome 11 (Figure 4A).

The genetic analysis of the F_2_ population of the NN1138-2 × *spwp* cross indicated that only one gene controls the phenotype of the *spwp* mutant under the genetic background of NN1138-2. To test which marker is linked to the mutant phenotype in the NN1138-2 × *spwp* population, all 111 F_2_-mutant individuals were genotyped using Sat_331 and Satt726 markers. The results showed that the SSR marker Satt726 on chromosome 14 was detected to be linked with the mutant phenotype, and then the *spwp1* locus was further delimited to a 1.44 Mb region between SSR markers S1382 and S1465 (Figure 3B). To isolate the *spwp1* and *spwp2* genes, 573 F_2:3_-mutant individuals derived from the heterozygous F_2_ plants of the KF1 × *spwp* cross were used for further fine-mapping. Under the polymorphic markers S1413, S1417, and S1418, two, zero, and seven recombination events were detected in the 573 F_2:3_-mutant individuals, respectively, indicating that the *spwp1* locus was pinpointed to the segment with a physical distance of approximately 183 kb between SSR markers S1413 and S1418 on chromosome 14 (Figure 3C). Additionally, under the polymorphic markers S1373, S1381, and S1385, one, zero, and three recombination events were detected, respectively, suggesting that the *spwp2* locus was mapped to a physical distance of an approximately 195 kb region on chromosome 11 between SSR markers S1373 and S1385 (Figure 4B). 

### 3.4. Whole-Genome Re-Sequencing Analysis

To rapidly identify the causal mutations, next-generation sequencing (NGS) was applied. Two DNA pool libraries were constructed and subsequently subjected to high-throughput whole-genome re-sequencing using the Illumina HiSeq 4000 platform. After filtering 102.49 G of raw data, 101.64 G of clean data was obtained for further analysis. The average Q20 ratio was ~98% and the Q30 ratio was ~94%, indicating the high quality of the sequencing data (Table S4). The numbers of clean reads were 163,984,765 for the wild type, with an average >31× and genome coverage >90.63%, and 168,202,480 for the mutant type, with an average >31× and genome coverage >90.62% (Table S5). A total of 2,046,994 SNPs and insertion–deletions (InDels) were obtained between the two re-sequenced samples for further analysis.

**Figure 5:**
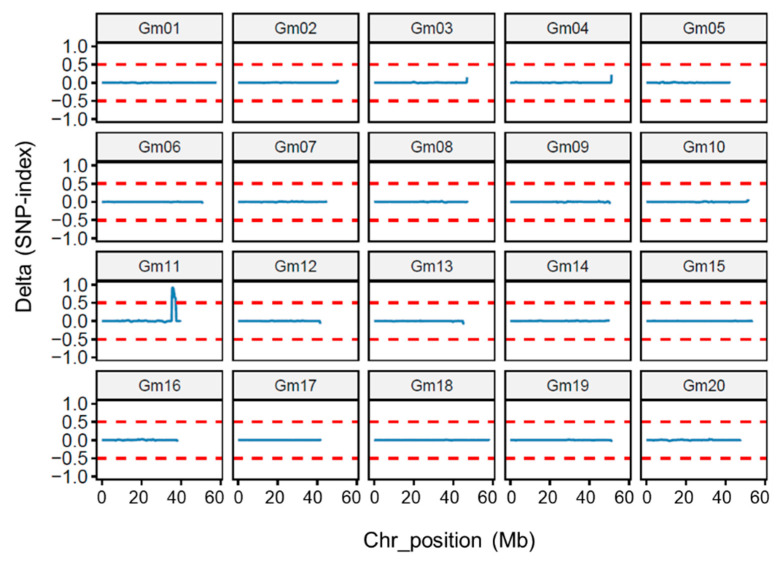
Distribution of delta SNP index values in 20 chromosomes of soybean. The x-axis indicates the physical position of chromosomes and the y-axis indicates delta SNP index values. Delta index = index (MT) − index (WT).

Because the near isogenic lines were selected from the offspring of a heterozygous single plant with a separation ratio of 3:1 (wild-type plants to mutant-type plants), only one pair of differential genes controlling short petioles and weakened pulvini traits exists in them, and the other gene is homozygous, i.e., the genotypes of the near isogenic lines are *SPWP1 SPWP1 spwp2 spwp2* vs. *spwp1 spwp1 spwp2 spwp2* or *spwp1 spwp1 SPWP2 SPWP2* vs. *spwp1 spwp1 spwp2 spwp2*. To determine the genotypes of near isogenic lines, we used the SNP index method to analyze the association for high-quality SNPs and InDels between the MT and WT DNA pools. The delta (SNP index) between the MT and WT across a 2 Mb window size was measured using a 50 kb step size and plotted for all 20 chromosomes of the soybean genome (Figure 5). Through analyzing the delta (SNP index) value, only one major peak was identified on chromosome 11 (Gm11), which suggests that the main differential loci of the near isogenic lines were located on chromosome 11, and therefore their genotypes should be *spwp1 spwp1 SPWP2 SPWP2* vs. *spwp1 spwp1 spwp2 spwp2*. The delta (SNP index) plotting, with a threshold of one, revealed that the interval with a physical distance of approximately 1.58 Mb on chromosome 11 between 36.26 Mb and 37.94 Mb was possibly linked to the phenotype of the *spwp* mutant. This positioning interval is consistent with the results of the linkage analysis, suggesting that the accuracy of the positioning results by the whole-genome re-sequencing analysis. 

### 3.5. RNA-Seq Analysis between the Spwp Mutant and WT

As another approach toward identifying the candidate genes and understanding the molecular mechanism underlying the *spwp* mutant’s phenotype, RNA-seq analysis was performed to investigate gene expression changes in the *spwp* mutant compared with the wild type. After filtering raw reads, an average of 41.53 million and 42.14 million clean reads were obtained from three WT samples and three MT samples, respectively. The percentages of Q30 were all above 93%, and the percentage of uniquely mapped reads ranged from 93.33% to 94.72%, indicating that the sequencing results were of high quality (Table S6).

**Figure 6:**
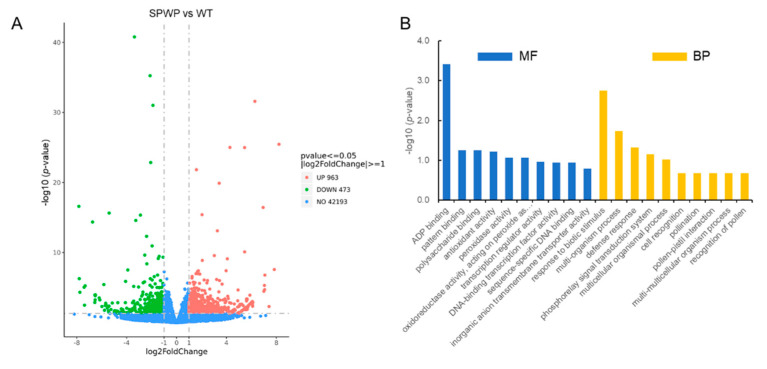
Differentially expressed genes (DEGs) in the *spwp* mutant compared with the wild-type (WT). (**A**): Volcano plot of DEGs determined by RNA-seq using the criteria *p*-value ≤ 0.05 and |log2 (Fold Change)| ≥ 1. Red dots indicate up-regulated genes and green dots indicate down-regulated genes. (**B**): enriched GO terms in the “molecular function” (MF) and “biological processes” (BP) categories of DEGs.

Compared with the WT, a total of 1436 differentially expressed genes (DEGs) were detected in the *spwp* mutant, including 963 up-regulated DEGs and 473 down-regulated DEGs (Figure 6A; Table S7). Gene Ontology (GO) analysis revealed that the DEGs were highly enriched for biological processes associated with “response to biotic stimulus”, “phosphorelay signal transduction system”, and “multi-organism process”, and were also enriched for molecular function categories related to “ADP binding”, “peroxidase activity”, and “polysaccharide binding” (Figure 6B). In addition, the Kyoto Encyclopedia of Genes and Genomes (KEGG) enrichment of DEGs showed that the “protein processing in endoplasmic reticulum”, “zeatin biosynthesis”, “brassinosteroid (BR) biosynthesis”, and “isoflavonoid biosynthesis” pathways were highly enriched (Figure S2).

### 3.6. Candidate Genes Analysis by Integrating Whole-Genome Re-Sequencing and RNA-Seq Data

Based on the annotations of the *Glycine max Wm82.a4.v1* genome database from SoyBase (http://www.soybase.org, accessed on 10 October 2021), 15/18 putative genes with annotated functions are predicted to reside in the *spwp1*/*spwp2* locus, respectively (Figure 3D, Table 2; Figure 4C, Table 3). 

The 18 genes located in the *spwp2* locus were screened for sequence variations in the coding DNA sequence between the WT and MT pool genome using the re-sequencing data. Compared with the WT pool genome, twelve SNPs were identified in the coding region of six candidate genes. Of these SNPs, ten formed missense substitutions in the amino acid sequence of five candidate genes (Table 4). These genes included: *CHX15* (*Glyma.11G229400*), a member of the sodium/hydrogen exchanger family; *Glyma.11g230000*, a member of the phosphoinositide-specific phospholipase C family; a transcription factor, *NF-X1* (*Glyma.11G230200*); and two homologous genes (*Glyma.11G230300* and *Glyma.11G230600*) that encode protein-kinase-domain-containing proteins. 

**Figure 7:**
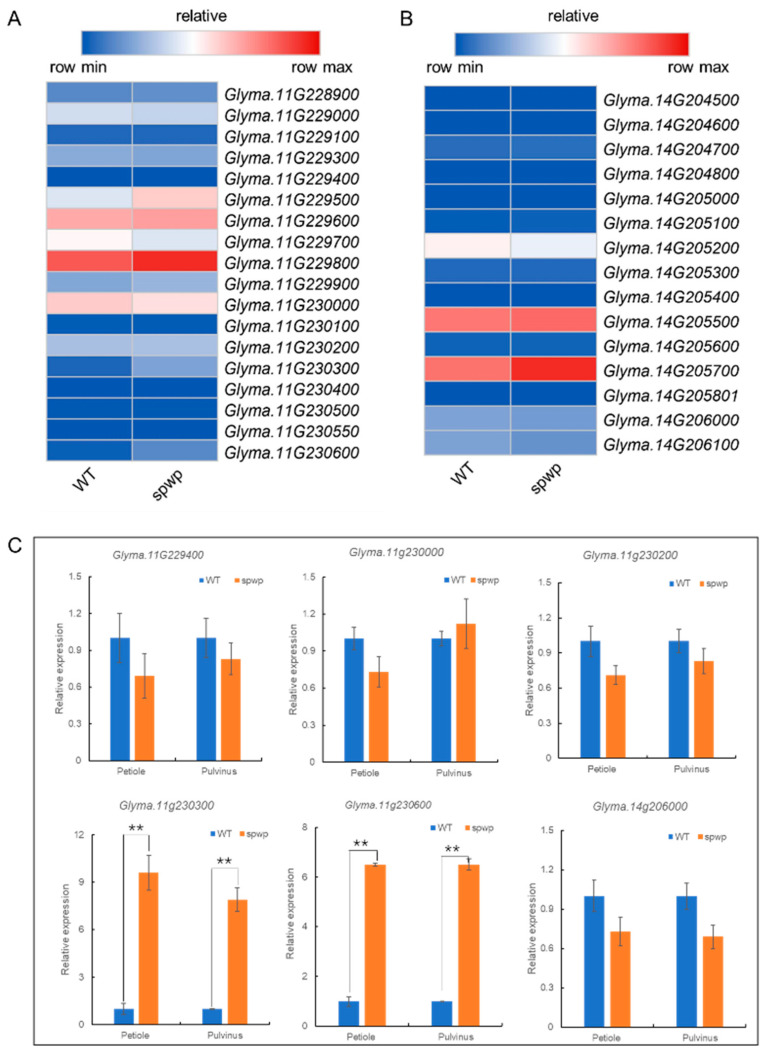
Analysis of the expression levels of all candidate genes using RNA-seq data, and six of these genes validated by RT-qPCR analysis. (**A**,**B**): Expression profiles of 18 and 15 genes predicted to be located in the *spwp2* locus (**A**) and the *spwp1* locus (**B**), respectively. The FPKM (fragments per kilobase of transcript per million mapped reads) value is the average of three biological replicates. (**C**): Relative expression levels of six genes detected by RT-qPCR in petioles and pulvini. Error bars indicate the SD (*n* = 3). Asterisks indicate a significant difference compared with the corresponding controls (**, *p* < 0.01; Student’s *t*-test).

Based on the synteny information obtained from SoyBase, the mapping region of *spwp1* and *spwp2* belongs to two nonhomologous genomic regions. Furthermore, according to the protein homolog information on Phytozome (https://phytozome-next.jgi.doe.gov/, accessed on 10 October 2021), no duplicated gene pairs were found between the *spwp1* locus and *spwp2* locus. Hence, *spwp1* and *spwp2* are two nonhomologous genes that likely have similar functions in regulating the development of petioles and pulvini. To further screen the candidate genes we analyzed the expression levels of all 33 predicted genes (15 genes from the *spwp1* locus and 18 genes from the *spwp2* locus) using the RNA-seq data. Only two DEGs (*Glyma.11g230300* and *Glyma.11g230600*) in the *spwp2* locus were significantly up-regulated in the *spwp* mutant (Figure 7A,B). It is interesting to note that *Glyma.11g230300* and *Glyma.11g230600* are homologous genes that encode protein-kinase-domain-containing proteins and that the functional annotation of the *Glyma.14g206000* gene located in the *spwp1* locus was also a “protein kinase domain”. To validate the RNA-seq analysis results we selected six genes (five genes having the SNPs mentioned above plus *Glyma.14g206000*) and performed a qRT-PCR analysis. The results of the qRT-PCR analysis showed the same expression trends as the RNA-seq data (Figure 7C). From the re-sequencing data of the MT pool, three mutation points were identified in the *Glyma.11g230300* gene, which all caused amino acid substitution. Moreover, one synonymous mutation and two missense point mutations were detected in the *Glyma.11g230600* gene (Table 3). To verify the results of the re-sequencing data and identify other sequence variations in the *Glyma.11g230300* and *Glyma.11g230600* genes, the coding sequences of these two candidate genes in the wild-type and mutant-type plants were amplified and sequenced. In addition to the SNPs identified by the NGS, two synonymous mutations and one missense point mutation were also detected in both genes, which led to a substitution of Ser with Arg in the *Glyma.11g230300* gene and a substitution of Ile with Thr in the *Glyma.11g230600* gene (Table S8). These results suggested that *Glyma.11g230300* and *Glyma.11g230600* may be the most important candidate genes for the *spwp2* locus, which is responsible for the short petioles and weakened pulvini phenotype of the *spwp* mutant in soybean.

### 3.7. Enrichment Analysis on DEGs Associated with the Phenotype of the Spwp Mutant

Notably, the GO analysis indicated that the DEGs were highly enriched in the GO term “phosphorelay signal transduction system” (Figure 6B). Among which, three homologous genes (*Glyma.03G130000*, *Glyma.11G155100*, and *Glyma.04G137600*) encoding response regulator receiver-domain-containing proteins homologous to Arabidopsis response regulator 9 (ARR9), two homologous genes (*Glyma.06G187000* and *Glyma.04G177900*) encoding a homologous protein of ARR6, and *Glyma.08G292400*, encoding a homologous protein of ARR3, were all upregulated. The ARRs together with histidine protein kinases such as CKI1 (*cytokinin-independent 1*) or CRE1 (*cytokinin response 1*) constitute two-component systems that play a central role in cytokinin signal transduction [34]. To et al. found that ARR5 and ARR6 function additively with ARR3 and ARR4 in the regulation of petiole elongation through the study of ARR multiple insertional mutants [35]. Moreover, the KEGG analysis showed that the DEGs were highly enriched in the “zeatin biosynthesis” pathway (Figure S2). The *Glyma.17G054500*, *Glyma.09G225400*, and *Glyma.03G133300* genes were all up-regulated in the *spwp* mutant, which are homologous to *cytokinin oxidase 3* (*CKX3*), *cytokinin oxidase 6* (*CKX6*), and *cytokinin oxidase 1* (*CKX1*) in Arabidopsis, respectively. The *CKX* genes are important regulators of active cytokinin levels, which catalyze the degradation of cytokinin [36]. These results showed that both the signal transduction and metabolism of cytokinin were altered in the *spwp* mutant. 

The plant hormone auxin controls diverse aspects of plant growth and development by regulating the fundamental cellular processes of expansion, division, and differentiation [37]. In the present study, we identified eighteen DEGs that are possibly involved in auxin signaling from the RNA-seq data (Table S9). Among them, two auxin efflux carrier genes, *PIN3A* (*Glyma.07G217900*) and *PIN3B* (*Glyma.20G014300*), were downregulated, while three genes (*Glyma.01G114000*, *Glyma.03G063600*, and *Glyma.03G063900*) encoding auxin-transporter-like proteins were upregulated. Except for polar transport, auxin signal transduction was also affected. The *Glyma.15G091000* gene encoding auxin response factor 8 (ARF8) was upregulated. Three Aux/IAA transcription factor genes (*Glyma.03G158700*, *Glyma.20G210400*, and *Glyma.13G356600*) were upregulated. Besides, the transcript levels of five SAUR genes were changed in the *spwp* mutant compared with WT.

In addition, we examined changes in the expression levels of transcription factors (TFs) based on the RNA-seq data. We identified 151 differentially expressed TFs belonging to 16 families, mainly including MYB (35), ZF (27), bHLH (21), AP2/EREBP (19), and WRKY (18) (Figure S3). Most of these families have previously been involved in plant growth and development. For example, overexpressing the *AtMYB96* gene can cause a dwarf phenotype with reduced lateral roots in Arabidopsis [38]. Two paralogous AtMYB124 (FOUR LIPS, FLP) and AtMYB88 proteins are required to limit cell divisions in the stomatal lineage [39]. In our study, we found that 26 genes encoding MYB TFs were up-regulated in the *spwp* mutant, while only nine genes were down-regulated. There are also 21 bHLH TF genes that are differently expressed in the *spwp* mutant compared with the WT. bHLH transcription factors play key roles in phytochrome signal transduction, organ development, and BR-responsive gene expression [40,41,42]. Recently, a study showed that AtLP1 (leaf-related protein 1) and AtLP2, two bHLH homologous proteins, regulate longitudinal cell elongation in Arabidopsis [43].

## 4. Discussion

### 4.1. Spwp Is a New Soybean Short Petiole Mutant with Weakened Pulvini

Short petioles are potentially useful for improving the per unit yield by altering the canopy profile and increasing planting density in soybean. Until now, many mutants with short petioles have been identified in soybean. For example, a previous study reported that the short petiole trait of *D76-1609* was controlled by a single recessive gene, *lps* [2]. The short petiole trait in *SS98206SP* was also controlled by a single recessive gene designated as *lps3*, which was mapped on chromosome 13 between SSR markers Sat_234 and Sct_033 [4,44]. The short petiole mutant *VP5* was identified from a fast neutron-induced mutant population, and the phenotype of the *VP5* mutant was co-segregated with an 837,919 bp deletion fragment on chromosome 17 [45]. Moreover, the *derived short petiole* (*dsp*) mutant was controlled by two recessive genes, *dsp1* and *dsp2*, which were mapped on chromosome seven with flanking markers BARCSOYSSR_07_0787 and BARCSOYSSR_07_0808 and on chromosome 11 between markers BARCSOYSSR_11_0037 and BARCSOYSSR_11_0043, respectively [46]. Recently, Wang et al. [47] found that the *rolled leaves and short petioles* mutant (*rlsp1*) was controlled by multiple genes, and identified 10 candidate regions on chromosomes three, six, eight, thirteen, and seventeen using sequencing-based bulked segregant analysis. The *Gmilpa1* mutant has shorter petioles and smaller pulvini compared to the wild type, and the underlying gene was isolated through map-based cloning approaches. It encodes an anaphase-promoting complex-like protein that appears to function by promoting cell growth and division [24]. Thus, the genetic regulation of petiole length is quite complex, and more efforts are needed to better understand the genetic basis of petiole length using new short-petiole genetic materials. In this study, we found that the *spwp* mutant was controlled by two recessive genes, *spwp1* and *spwp2*, which were, respectively, mapped in a 183 kb genomic region on chromosome 14 and in a 195 kb genomic region on chromosome 11 by using the traditional map-based cloning method (Figure 3A–D and Figure 4A–C). To our knowledge, no genes related to the length of leaf petioles have been reported in those two regions. Hence, *spwp* is a novel short-petiole mutant controlled by two genes.

Pulvini show nyctinastic movement through turgor pressure changes in their motor cells [15]. In the present study, we found that the pulvini of the *spwp* mutant were incomplete and that the nyctinastic motions of pulvini and leaflets were both diminished in the *spwp* mutant. Furthermore, the paraffin section observation results showed that the number and size of motor cells on the abaxial and adaxial sites were reduced in the pulvini of the *spwp* mutant (Figure 2C,D). Therefore, the defects in nyctinastic mobility in the *spwp* mutant were likely due to the under-developed pulvini. Although these results are similar to the previous characterization of the soybean *Gmilpa1* mutant [24], the developmental defects in the pulvini are caused by different genes. Therefore, this study provides a new genetic resource for soybean ideotype breeding and understanding pulvinus development.

### 4.2. Identification Candidate Genes of the Spwp Mutant through Whole-Genome Re-Sequencing and RNA-Seq

The phenotype of the *spwp* mutant was controlled by two genes, *spwp1* and *spwp2*, which were mapped to two nonhomologous fragments on chromosome 14 and chromosome 11, respectively. Fifteen genes were predicated within the *spwp1* locus. Of these genes, five homologous genes contain the NB-ARC domain, and a recent study found that *Rsc4-3* (*Glyma.14G204700*) confers soybean resistance to soybean mosaic virus [48]. Three of these genes had no gene annotation and their expression levels were not detected in the RNA-seq data (Table 2; Figure 7B). This being the case, these three genes may have no function. *Glyma.14g206000* is considered to be the most likely candidate gene for the *spwp1* locus that encodes a protein with a protein kinase domain. However, no variations were found in the coding sequences of *Glyma.14g206000*, and its expression level was also not significantly different between the mutant-type and wild-type plants (Figure 7C). Eighteen genes were predicated within the *spwp2* locus, and the functional annotations of only one gene are unknown (Table 3). *Glyma.11g230300* and *Glyma.11g230600* were selected as the most important candidate genes for governing the short petioles and weakened pulvini phenotype of the *spwp* mutant due to their coding sequences and gene expression levels, which both changed between the *spwp* mutant and the WT, based on whole-genome re-sequencing as well as RNA-seq analysis and further confirmed by Sanger sequencing and RT-qPCR (Figure 7A,C; Table 4). Intriguingly, those two genes are homologous and both encode a protein-kinase-domain-containing protein homologous to Arabidopsis CRINKLY 4 RELATED 3 (CCR3 or CRR3), which belongs to the CRINKLY4 (CR4) family of receptor-like kinases. CR4 was first identified in maize, where the *cr4* mutation affects leaf epidermis differentiation [49]. The following research shows that CR4 play an important role in multiple aspects of plant growth and development. For example, ARABIDOPSIS CRINKLY 4 (ACR4) is essential for epidermal cell differentiation in leaves and seed coats [50], cell layer organization during ovule integument and sepal margin development [51], and proper embryogenesis [52]. OsCR4 is required to maintain the interlocking of the palea and lemma [53], and positively regulates culm elongation in rice [54]. Moreover, ACR4 is part of a mechanism controlling formative cell divisions in the Arabidopsis root [55,56]. Some studies have suggested that the ACR4 functions as a coreceptor of other receptors, such as CLV3 INSENSITIVE KINASES (CIKs) and CLAVATA1 (CLV1), to mediate CLE40 signaling, and that the CLE40-ACR4-WOX5 signaling pathway plays a central role in maintaining the stem cell niche and in controlling columella cell development [57,58,59,60]. It has been proven that WOX5 is involved in cytokinin signaling and auxin signaling pathways [61,62,63].

In the present study, our RNA-seq data showed that the expression levels of DEGs related to cytokinin signal transduction and metabolism were altered in the *spwp* mutant compared with the WT (Figure 6B; Table S2). Moreover, a subset of DEGs involved in the auxin signaling transduction network were also detected in the *spwp* mutant (Table S9). The results of the paraffin section show that cell expansion and cell division were affected in the leaf petioles and pulvini of the *spwp* mutant (Figure 1C,D and Figure 2C,D). These results are reminiscent of the candidate genes *Glyma.11g230300* and *Glyma.11g230600*, which may be involved in cytokinin and auxin signal transduction pathways to regulate cell division and cell expansion in the leaf petioles and pulvini of the *spwp* mutant. However, we cannot rule out the possibility of other genes in the positioning interval responsible for the *spwp* mutant phenotype, and more work needs to be done in the future to validate the candidate genes. It is possible to dissect the reasons of *spwp* mutant formation using the proteomics and metabolomics strategies as well as integrate transcriptomics with them to elaborate on the regulatory networks of petiole and pulvinus growth and development in different levels.

### 4.3. The Spwp Mutant Also Showed Defective Leaf Movement

In legume species, a common phenomenon is that leaves open during the day and fold at night, which is diurnal leaf movement named nyctinasty. Structurally, nyctinastic movement is driven by the pulvinus, a specialized motor organ located at the base of leaves and leaflets [21]. In this study, we found that the *spwp* mutant also showed defective leaf movement, that is, leaflets remained in a horizontal (open) position during day and night. Several factors have been reported to influence the leaf movement. For example, the F-box protein MIO1/SLB1, a component of the SKP1/Cullin/F-box (SCF) E3 ubiquitin ligase complex, influences leaf movement through possibly controlling the length of the pulvinus [64]. Aquaporins, anion channels, and K^+^ channels are thought to regulate volume changes of the motor cells and thus control leaf movement [18,65,66]. Interestingly, a recent study showed that the geometry of the compound leaf is important for leaf movement in *Medicago truncatula* [67]. Moreover, the plant hormone BR homeostasis is critical for nyctinastic leaf movement but has no effect on pulvinus determination [68]. In this study, the KEGG pathway enrichment analysis showed that the DEGs were highly enriched in BR biosynthesis (Figure S2). Among them, the transcript levels of *Glyma.08G193900* and *Glyma.14G059900* were up-regulated in the *spwp* mutant. The *Glyma.14G059900* gene is homologous to the Arabidopsis gene *AT5G05690*, which encodes a cytochrome P450 monooxygenase that converts 6-deoxocathasterone into 6-deoxoteasterone in the late C6 oxidation pathway of brassinolide biosynthesis [69]. By contrast, the transcript level of *Glyma.17G118100* was down-regulated, which is homologous to *AT2G01190* (*PHYB ACTIVATION TAGGED SUPPRESSOR 1*, *BAS1*) in Arabidopsis. BAS1 is thought to be a control point between multiple photoreceptor signal transduction pathways and BR signaling [70,71]. In addition, *GmBRI1b* (*Glyma.04g218300*) was down-regulated in the *spwp* mutant. GmBRI1b, a homolog of AtBRI1, has been shown to function as a BR receptor [72]. These results suggested that not only BR biosynthesis but also BR signal transduction were altered in the *spwp* mutant, which was likely responsible for the defective leaf movement of the *spwp* mutant.

## Figures and Tables

**Table 1:** Genetic analysis of the *spwp* mutant in the F_2_ populations.

F_2_ Populations	No. of Total Plants	No. of WT Plants	No. of MT Plants	Expected Ratio	χ^2^_0.05_	*p*-Value
KF1 × *spwp*	692	650	42	15:1	0.02	0.87
NN1138-2 × *spwp*	540	420	120	3:1	2.22	0.14

**Table 2:** Functional annotation of candidate genes in the *spwp1* mapping interval.

NO.	Gene Name	Start (bp)	Stop (bp)	Annotation
1	*Glyma.14g204500*	47796602	47808011	NB-ARC domain
2	*Glyma.14g204600*	47810987	47824929	NB-ARC domain
3	*Glyma.14g204700*	47831269	47846928	NB-ARC domain
4	*Glyma.14g204800*	47834239	47834920	No annotation
5	*Glyma.14g205000*	47855678	47868591	NB-ARC domain
6	*Glyma.14g205100*	47870805	47877043	KIP1-like protein
7	*Glyma.14g205200*	47892074	47896155	Cytochrome P450
8	*Glyma.14g205300*	47895257	47907012	NB-ARC domain
9	*Glyma.14g205400*	47909453	47911194	No annotation
10	*Glyma.14g205500*	47911849	47919011	Domain found in IF2B/IF5
11	*Glyma.14g205600*	47929529	47931437	AP2 domain
12	*Glyma.14g205700*	47936205	47940755	Ubiquitin-conjugating enzyme
13	*Glyma.14g205801*	47939508	47939654	No annotation
14	*Glyma.14g206000*	47946254	47952120	Protein kinase domain
15	*Glyma.14g206100*	47972022	47975414	BTB/POZ domain/NPH3 family

**Table 3:** Functional annotation of candidate genes in the *spwp2* mapping interval.

NO.	Gene Name	Start (bp)	Stop (bp)	Annotation
1	*Glyma.11g228900*	37308090	37313815	Cytochrome P450
2	*Glyma.11g229000*	37315051	37320857	Nuclear pore component
3	*Glyma.11g229100*	37328504	37331716	Cholesterol-capturing domain
4	*Glyma.11g229300*	37343716	37352599	ABC1 family
5	*Glyma.11g229400*	37352888	37356662	Sodium/hydrogen exchanger family
6	*Glyma.11g229500*	37376823	37382293	GDSL-like lipase/acylhydrolase
7	*Glyma.11g229600*	37385109	37391331	GINS complex protein
8	*Glyma.11g229700*	37394752	37395822	Protein of unknown function (DUF1313)
9	*Glyma.11g229800*	37396251	37399628	Cyclophilin type peptidyl-prolyl cis-trans isomerase/CLD
10	*Glyma.11g229900*	37404283	37408842	Phosphatidylinositol-specific phospholipase C, X domain
11	*Glyma.11g230000*	37410777	37417244	Phosphatidylinositol-specific phospholipase C, X domain
12	*Glyma.11g230100*	37419547	37424310	Phosphatidylinositol-specific phospholipase C, X domain
13	*Glyma.11g230200*	37425247	37433034	NF-X1-type zinc finger
14	*Glyma.11g230300*	37433772	37435676	Protein kinase domain
15	*Glyma.11g230400*	37443779	37446784	Protein kinase domain
16	*Glyma.11g230500*	37461012	37462619	Protein kinase domain
17	*Glyma.11g230550*	37464702	37464827	Protein kinase domain
18	*Glyma.11g230600*	37468983	37471869	Protein kinase domain

**Table 4:** The SNPs in the *spwp2* locus on chromosome 11 in the *spwp* mutant.

Genes	Physical Position in Chromosome 11 (bp) ^a^	Mutation ^b^	Type	Conversion of AA
*Glyma.11g229000*	37318335	C→T	Synonymous	/
*Glyma.11g229400*	37354075	A→G	Nonsynonymous	Glu→Gly
*Glyma.11g230000*	37411111	G→C	Nonsynonymous	Gly→Ala
	37411209	G→C	Nonsynonymous	Val→Leu
*Glyma.11g230200*	37430249	G→C	Nonsynonymous	Gly→Ala
	37431363	A→T	Nonsynonymous	Gln→His
*Glyma.11g230300*	37433861	G→C	Nonsynonymous	Val→Leu
	37433873	T→C	Nonsynonymous	Tyr→His
	37435019	G→C	Nonsynonymous	Ala→Pro
*Glyma.11g230600*	37469232	T→C	Nonsynonymous	Val→Aal
	37469248	T→C	Synonymous	/
	37470305	A→C	Nonsynonymous	Thr→Pro

^a^ represents the physical position in the Williams 82 reference genome (*Glycine max Wm82.a4.v1*). ^b^ represents the genotypes of the corresponding mutated locus present in the MT pool genome compared with the WT pool genome.

## Data Availability

The data presented in this study are available in the article and supplementary material.

## Supplementary Materials

The following supporting information can be downloaded at: https://www.mdpi.com/article/10.3390/genes13020185/s1, Figure S1: Comparison of the leaf petiole angle between the wild-type plants (WT) and the mutant-type plants (MT); Figure S2: The scatter diagram of KEGG enrichment; Figure S3: The expression analysis of differentially expressed transcription factors (TFs) based on the RNA-seq data; Table S1: The primers used for mapping the *spwp1* locus; Table S2: The primers used for mapping the *spwp2* locus; Table S3: List of the primers used for the qRT-PCR analysis of six candidate genes; Table S4: Summary of re-sequencing data quality of the wild-type and mutant-type DNA pools; Table S5: Mapping information for the wild-type and mutant-type DNA pools by whole-genome re-sequencing; Table S6: Throughput and quality of RNA-seq of the *spwp* mutant and the wild type (WT); Table S7: The differentially expressed genes between the *spwp* mutant and the wild type (WT); Table S8: The SNPs in the candidate genes detected by Sanger sequencing; Table S9: Auxin-related genes differentially expressed between the *spwp* mutant and the wild type (WT).

## Abbreviations

RNA-seq	RNA sequencing
AUX/IAA	Auxin/indoleacetic acid
SAUR	SMALL AUXIN-UP RNA
PIN3A	PIN-FORMED 3A
PIN3B	PIN-FORMED 3B
bHLH	Basic helix–loop–helix protein
WRKY	Transcription factor (TF), partake WRKYGQK heptapeptide at N-terminal
MYB	Myeloblastosis (avian myeloblastosis viral oncogene homolog) TF
ZF	Zinc finger TF
AP2/EREBP	Apetala2/ethylene-responsive element binding protein
NB-ARC	Nucleotide-binding domain shared with APAF-1, various R proteins, and CED-4
CLE40	CLV3/EMBRYO SURROUNDING REGION (ESR) 40
WOX5	WUSCHEL-RELATED HOMEOBOX5
