# Supplementary material for: Combining Fine Mapping, Whole-Genome Re-Sequencing, and RNA-Seq Unravels Candidate Genes for a Soybean Mutant with Short Petioles and Weakened Pulvini
Source: Genes (Basel). 2022 Jan 21;13(2):185. doi: 10.3390/genes13020185 (PMC8872139; doi:10.3390/genes13020185)
Supplement: Supplementary file 1 [file genes-13-00185-s001.zip › Supplementary materials (Figure S1-S3 and Table S1-S6, S8-S9) .pdf]

Supplementary Materials

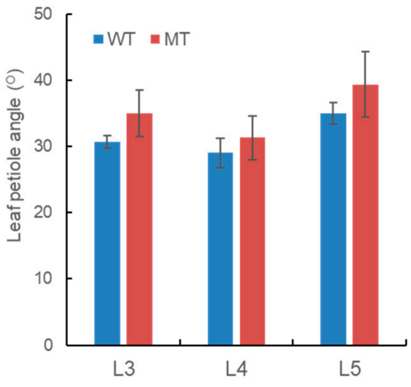

Figure S1. Comparison of the leaf petiole angle between wild-type plants (WT) and mutant-type plants (MT). L3, L4, L5 represented the third to fifth leaf petiole from top to bottom, respectively.

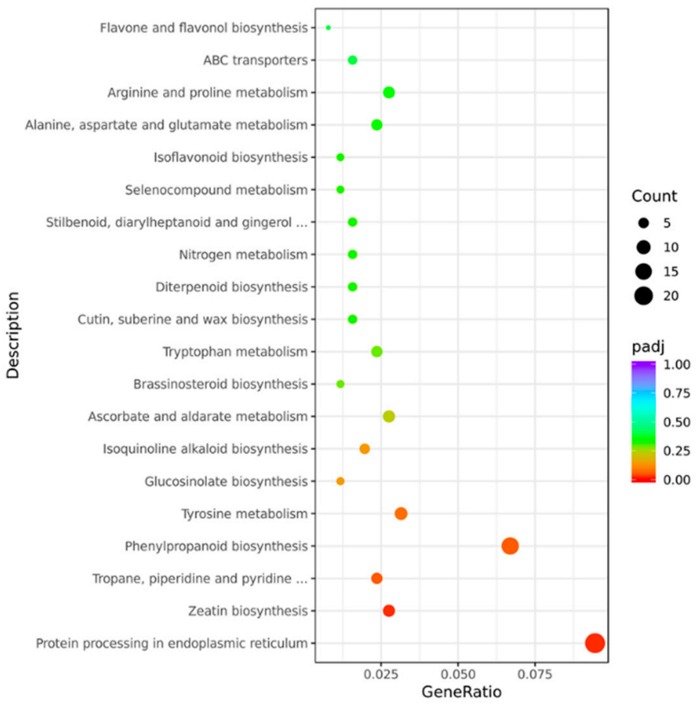

Figure S2. The scatter diagram of Kyoto Encyclopedia of Genes and Genomes (KEGG) enrichment. The GeneRatio means the ratio of the differentially expressed genes (DEGs) number enriched in a pathway to the DEGs number enriched in all pathways. The size of the dot indicates the number of genes and the color of the dot corresponds to the different padj ranges.

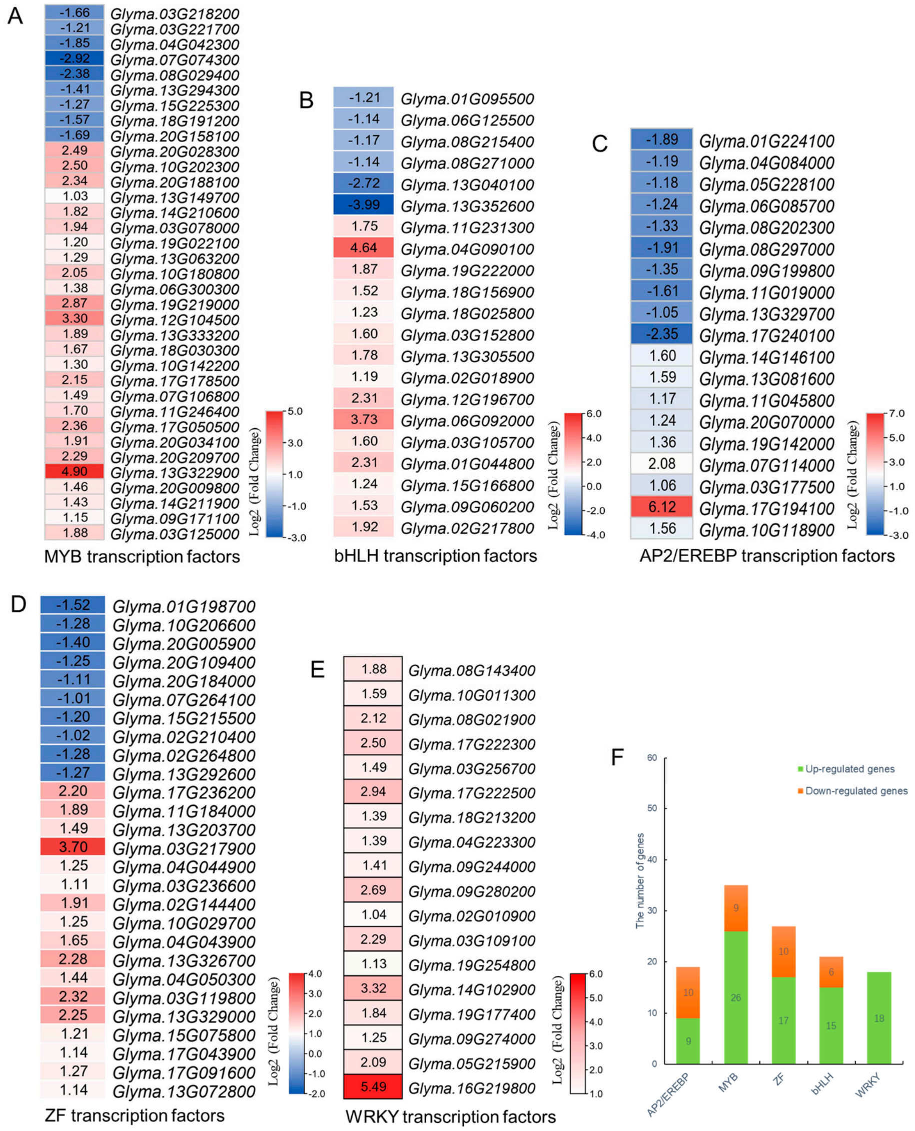

Figure S3. The expression analysis of differentially expressed transcription factors (TFs) based on the RNA-seq data.

(**A**–**E**), down-regulated and up-regulated differentially expressed genes (DEGs) in MYB (**A**), bHLH (**B**), AP2/EREBP (**C**), ZF (**D**), WRKY transcription factor families (**E**). (**F**), distribution of DEGs in different TF families.

Table S1. The primers used for mapping *spwp1* locus.

SSR Marker	Short name	Forward primer sequences (5'-3')	Reverse primer sequences (5'-3')
Satt063	/	AAATGATTAACAATGTTTATGAT	ACTTGCATCAGTTAATAACAA
BARCSOYSSR_14_1367	S1367	TGGTTTAGCTACTCATTTTGTCTTT	TGCATACACCTAAAATCACTTGTT
BARCSOYSSR_14_1373	S1373	TGATGGAACCAATTCACAGAAA	TGCCCTTGTGTGTACCTTCA
BARCSOYSSR_14_1375	S1375	TGACATGTGGTGAAGGAGGA	AGCCTGCAGAAAATTGGAAA
BARCSOYSSR_14_1382	S1382	GCGTTTTTAGTATGGATAATGTTTT	GCGAAGGGACAAGAGTGAT
BARCSOYSSR_14_1390	S1390	CCAACGAACATAGGGGTGAT	GAGTTTCGGAGACCTCAACG
BARCSOYSSR_14_1404	S1404	CACACCCCTTTTGTGCTTTT	AAGCCTGCAAGAGAATCCAA
BARCSOYSSR_14_1413	S1413	CCTCGCGGTTCGTTTATTAT	CAGTTTCAATGCTGTGCATTTT
BARCSOYSSR_14_1418	S1418	CACACAATATTTTGGGAATTTTATCA	TGTTTTGGTTCTTAACACTTTCACA
BARCSOYSSR_14_1421	S1421	GCGATCGTGCAAGAAAATA	GCGGTGGACTTCGCCTCAAATAAT
BARCSOYSSR_14_1423	S1423	TCAGAACAGATTCGCTCCCT	GGTCTCCGAGAATTGGATCA
BARCSOYSSR_14_1455	S1455	AATTGATCTCTGTCGCCCAA	TGTTCCACGTTATTTTTGCG
BARCSOYSSR_14_1465	S1465	CACACCCTAGAAAAGGGCAA	CCATCACAACGGAACTCAAA
BARCSOYSSR_14_1498	S1498	CGCCTACTAAGTGGATGATGC	CGGTTCGACTTTTAACCACC

Table S2. The primers used for mapping *spwp2* locus.

SSR Marker	Short names	Forward primer sequences (5'-3')	Reverse primer sequences (5'-3')
Sat_095	/	TGGACACGTTGAAAAGAGTAAAAAGTT	CAGGCCCAAAGTAAATCTGAAAAA
BARCSOYSSR_11_1335	S1335	CAATTTTCTCTATTTCGATCCATTT	ACTTCACGTATTATATTTTTCAATGTG
BARCSOYSSR_11_1344	S1344	TTTGATACCGACACCCCTTC	TCTTTTTGCTTATGATTAATGTTCC
BARCSOYSSR_11_1360	S1360	CGAAGCTTATGACTAGGGTGGA	CAAAAGCAAAGAGTCTATGTCGC
BARCSOYSSR_11_1373	S1373	GCGAGAAAATAATCCTGCTCAAG	GCGTTTAAGTCCAATAACAAAGATAAC
BARCSOYSSR_11_1385	S1385	CCTCCCCTTTTGGTGAGTAA	TTATGCCTTTTCCCAACAGC
BARCSOYSSR_11_1390	S1390	ACGATGGCATTGCAGTCTTT	CAATATATGCAGGGGAAGGG
BARCSOYSSR_11_1398	S1398	TGCTTGAGGAGTGGATCCTT	CGATTCTTCAAACCAGTGCC
BARCSOYSSR_11_1406	S1406	CGTAAGCCCTCAACACCATT	AAATTAGGCATCGACTAGGGC
BARCSOYSSR_11_1445	S1445	TGCTCCACCACCTACATCAA	GTGTTTTCCTGCTGCTCCTC
Sat_331	/	GCGGTGAATATCCATCAGCCATGAAATTATA	GCGTGCCCATTATTTGTTGGATATTTGTTTG
BARCSOYSSR_11_1481	S1481	AAAATAGTGGTGCGACCAAA	CGGCTGAAGTACTGGGAAGT

Table S3. List the primers used for qRT-PCR analysis of six candidate genes.

Gene ID	Product Size (bp)	Forward strand sequence (5′–3′)<br>Reverse strand sequence (5′–3′)	Illustration
Glyma.11G229400	142	GTTAGCCATGGTGCTAGACAG<br>TCGAGGAATATCATCACGACAACG	for qRT-PCR
Glyma.11g230000	175	ATGGCACTGTTCTTATGCCCT<br>TGTGACTGGCCATAATCGTGT	
Glyma.11G230200	175	GCTCTGAGATATGCGGCAG<br>GGACATCTAGGTGGCTTATTGT	
Glyma.11G230300	155	GGAGTGAGACTGGGTCCATTT<br>ACTCCTCATCGCCCTGTCTT	
Glyma.11G230600	155	CGATGTGAATGAGGCCGAGG<br>TGGAAGTGCTGTCATGGCTA	
Glyma.14g206000	102	CCCTCTACACCTCCTCCATCGT<br>CGCAATTGCAATGATAGCTGCC	
Glyma.18G290800	142	CGGTGGTTCTATCTTGGCATC<br>GTCTTTCGCTTCAATAACCCTA	reference gene (*Actin11*)

Table S4. Summary of re-sequencing data quality of wild type and mutant type DNA pools.

Samples	Raw Base(G)	Clean Base(G)	Effective Rate(%)	Error Rate(%)	Q20(%)	Q30(%)	GC Content(%)
Wild type	50.58	50.17	99.18	0.03	98.29	94.71	35.88
Mutant type	51.91	51.47	99.16	0.03	98.25	94.6	35.66

Table S5. Mapping information for wild type and mutant type DNA pools by whole genome re-sequencing.

Samples	Raw Reads	No. of total clean read pairs	Mapped rate (%)	Average depth (x)	Genomic coverage (%)
Wild type	168,593,500	163,984,765	99.26	~31x	90.63
Mutant type	173,032,393	168,202,480	97.80	~31x	90.62

Table S6. Throughput and quality of RNA-seq of the *spwp* mutant and wild type (WT).

sample	raw_reads	clean_reads	unique_map	error_rate	Q20(%)	Q30(%)	GC(%)
WT_1	40317276	38920798	36324019(93.33%)	0.03	97.61	93.36	44.68
WT_2	45026344	43527798	41016346(94.23%)	0.03	97.77	93.6	44.01
WT_3	44987034	42132182	39421396(93.57%)	0.03	97.76	93.65	44.59
spwp_1	40175250	39138766	36780942(93.98%)	0.03	97.6	93.27	44.14
spwp_2	44800474	43836990	41417538(94.48%)	0.03	97.72	93.48	43.65
spwp_3	44270438	43446476	41152245(94.72%)	0.03	97.79	93.64	43.18

Table S8. The SNPs in the candidate genes detected by the Sanger sequencing.

Gene	Physical position in chromosome 11 (bp)	Mutation	Type	Conversion of AA
Glyma.11g230300	37434340	T→A	Synonymous	/
	37434391	T→A	Synonymous	/
	37434392	A→C	Nonsynonymous	Ser→Arg
Glyma.11g230600	37470826	G→A	Synonymous	/
	37470919	T→A	Synonymous	/
	37471188	T→C	Nonsynonymous	Ile→Thr

Table S9. Auxin-related genes differentially expressed between the *spwp* mutant and wild type (WT).

Number	Gene Name	Annotation Description	log2FoldChange	*spwp*[a]	WT[b]	*p*-value
1	Glyma.03G158700	AUX/IAA family (IAA14)	1.95	1283.13	333.10	0.021309886
2	Glyma.20G210400	AUX/IAA family (IAA14)	2.08	97.30	23.00	0.004074022
3	Glyma.13G356600	AUX/IAA family (IAA27)	1.03	150.66	73.98	0.032051363
4	Glyma.07G217900	Auxin efflux carrier family protein (PIN3A)	-1.27	660.70	1591.54	0.001841378
5	Glyma.20G014300	Auxin efflux carrier family protein (PIN3B)	-1.25	378.07	897.59	0.001257736
6	Glyma.15G091000	auxin response factor 8 (ARF8)	1.00	1923.60	960.91	0.020643902
7	Glyma.01G114000	auxin transporter-like protein 1 (LAX1)	1.15	130.34	58.93	0.014096051
8	Glyma.03G063600	auxin transporter-like protein 3 (LAX3)	1.43	620.80	230.62	0.000568943
9	Glyma.03G063900	auxin transporter-like protein 4 (LAX4)	1.90	59.89	16.05	0.033911735
10	Glyma.19G161100	Auxin-regulated protein 28 (AUX28)	3.39	201.64	19.22	4.08E-05
11	Glyma.07G043000	Dormancy/auxin associated protein	2.73	13.12	1.97	0.036479849
12	Glyma.16G011400	Dormancy/auxin associated protein	1.74	106.09	31.96	0.009081428
13	Glyma.06G115100	IAA-leucine resistant (ILR)-like gene 6 (ILL6)	-1.26	90.45	216.09	0.000155564
14	Glyma.04G006400	SAUR-like auxin-responsive protein family (SAUR23)	-4.62	0.00	4.45	0.046521288
15	Glyma.19G183700	SAUR-like auxin-responsive protein family (SAUR48)	-1.65	16.33	51.13	0.012042824
16	Glyma.16G129600	SAUR-like auxin-responsive protein family (SAUR50)	2.16	12.06	2.71	0.046993887
17	Glyma.15G182300	SAUR-like auxin-responsive protein family (SAUR70)	3.86	14.66	1.02	0.005671911
18	Glyma.17G046000	SAUR-like auxin-responsive protein family (SAUR78)	1.63	78.72	25.54	0.019125912

[a, b] represent the mean readcount value of three *spwp* mutant-type (*spwp*) and wild-type (WT) samples after standardization, respectively.
